# Supplementary material for: Comparison of tests done, and tuberculosis cases detected by Xpert® MTB/RIF and Xpert® MTB/RIF-Ultra in Uganda
Source: PLoS One. 2022 Oct 10;17(10):e0275960. doi: 10.1371/journal.pone.0275960 (PMC9550068; doi:10.1371/journal.pone.0275960)
Supplement: Attachment — Submitted filename: Response to reviewers PONE-D-20-34522R1.doc [file pone.0275960.s002.doc]

April 19^th^, 2022

The Academic Editor
PLoS ONE

Dear Academic Editor,

**RE: Response to the editor and reviewers' comments on Manuscript #: PONE-D-20-34522R1: “Comparison of tests done and Tuberculosis cases detected by Xpert MTB/RIF and Xpert MTB/RIF Ultra in Uganda”.**

First and foremost, we would like to express our gratitude for allowing us to revise our manuscript for possible publication in your prestigious journal, *PLoS ONE.* We would also like to thank the editor and reviewers for taking the time to provide enriching scientific comments.

We have now revised the paper in line with the editor’s and reviewers’ comments and suggestions and believe that it is now a much more improved paper than the version we originally submitted.

We will be glad to provide any further explanations on the changes made in the paper, should this be necessary. Otherwise, we look forward to having our paper accepted for publication in *PLoS ONE*.

Regards

Michael Kakinda MD, MPH

Corresponding Author

**POINT-BY-POINT RESPONSE TO THE EDITOR’S AND THE REVIEWERS’ COMMENTS**

**Response to the Editor’s comments**

In this manuscript Xpert MTB/RIF has been compared with Xpert MTB/RIF Ultra by including the data from 112 sites during two different periods. Although it has been concluded that there was no significant difference in test performance and overall detection of DS-TB and RR-TB, the study could have highlighted the difference between smear positive and negative samples. Secondly, it would have been interesting to note whether there were more cases from any extrapulmonary sample. There have been studies that have shown that sensitivity and specificity varied according to the type of sample. Thirdly, the performance of Xpert MTB/RIF ultra in the pediatric age group could have been shown.

We thank the editor for this comment since it is fundamental to why the Xpert MTB/RIF was introduced. The authors have tried to accommodate the comment in the limitations section since it is not routine to have a culture for all Sputum TB samples. Unless they are Rifampicin Resistant Indeterminate. The same applies to the extra-pulmonary versus the pulmonary samples, the former samples are likely to be negligible. So, there are unlikely to change the overall results.

Line 75- The sentence could be completed after 'Jan2012'

We have made this correction.

Line 110- Rifampicin resistant TB should be RR-TB

This correction has been made.

4th and 5th lines under 'Comparison between Xpert® MTB/RIF and Xpert® MTB/RIF Ultra'- MDR-TB to be replaced by RR-TB. Also, in Table 2, MDR-TB is to be replaced by RR-TB.

This correction has been made, see lines 4 and 5 and Table 2.

1. Please ensure that your manuscript meets PLOS ONE's style requirements, including those for file naming.

**Response**: We have revised our manuscript to ensure that the level 1 headings are 18 pt font, while level two headings are 16 pt font and level 3 are 14 pt font. They are all in bold and we have capitalized only the first word of the heading, the first word of the sub-heading and any proper nouns and genus names. The rest of the text is 12 pt font. Supplementary information files have been renamed following the PLOS ONE’s style requirements.

2. We suggest you thoroughly copyedit your manuscript for language usage, spelling, and grammar. If you do not know anyone who can help you do this, you may wish to consider employing a professional scientific editing service.  

Whilst you may use any professional scientific editing service of your choice, PLOS has partnered with both American Journal Experts (AJE) and Editage to provide discounted services to PLOS authors. Both organizations have experience helping authors meet PLOS guidelines and can provide language editing, translation, manuscript formatting, and figure formatting to ensure your manuscript meets our submission guidelines. To take advantage of our partnership with AJE, visit the AJE website (http://learn.aje.com/plos/) for a 15% discount off AJE services. To take advantage of our partnership with Editage, visit the Editage website (www.editage.com) and enter referral code PLOSEDIT for a 15% discount off Editage services.  If the PLOS editorial team finds any language issues in text that either AJE or Editage has edited, the service provider will re-edit the text for free.

Upon resubmission, please provide the following:

- The name of the colleague or the details of the professional service that edited your manuscript
- A copy of your manuscript showing your changes by either highlighting them or using track changes (uploaded as a *supporting information* file)
- A clean copy of the edited manuscript (uploaded as the new *manuscript* file)

The authors have edited the manuscript using commercially available software editors, and all the authors did edit the manuscript. We have provided a manuscript with tracked changes and a clean copy of the edited manuscript.

3. You indicated that ethical approval was not necessary for your study. We understand that the framework for ethical oversight requirements for studies of this type may differ depending on the setting and we would appreciate some further clarification regarding your research. Could you please provide further details on why your study is exempt from the need for approval and confirmation from your institutional review board or research ethics committee (e.g., in the form of a letter or email correspondence) that ethics review was not necessary for this study? Please include a copy of the correspondence as an "Other" file.

We have made changes for the ethical approval section. I have attached a letter from the Operational Research and Ethics Review Committee of the National Tuberculosis and Leprosy Program of Uganda. The authors had previously got the approval to use the data and publish it but, it had not been written. However, we got a copy and will attach it under others.

4. In your Data Availability statement, you have not specified where the minimal data set underlying the results described in your manuscript can be found. PLOS defines a study's minimal data set as the underlying data used to reach the conclusions drawn in the manuscript and any additional data required to replicate the reported study findings in their entirety. All PLOS journals require that the minimal data set be made fully available. For more information about our data policy, please see http://journals.plos.org/plosone/s/data-availability.

Upon re-submitting your revised manuscript, please upload your study’s minimal underlying data set as either Supporting Information files or to a stable, public repository and include the relevant URLs, DOIs, or accession numbers within your revised cover letter. For a list of acceptable repositories, please see http://journals.plos.org/plosone/s/data-availability#loc-recommended-repositories. Any potentially identifying patient information must be fully anonymized.

**Response**: The following statement has been used to meet the ‘Data availability’ requirements of the journal:

“**All relevant data are within the paper and its Supporting Information files**”

5. Thank you for stating the following financial disclosure:

"No"

At this time, please address the following queries:

a. Please clarify the sources of funding (financial or material support) for your study. List the grants or organizations that supported your study, including funding received from your institution.
b. State what role the funders took in the study. If the funders had no role in your study, please state: “The funders had no role in study design, data collection, and analysis, decision to publish, or preparation of the manuscript.”
c. If any authors received a salary from any of your funders, please state which authors and which funders.
d. If you did not receive any funding for this study, please state: “The authors received no specific funding for this work.”

 Please include your amended statements within your cover letter; we will change the online submission form on your behalf.

**Response**: The following financial disclosure information statement should be included in the online submission form:

“There was no grant or funds provided for this study”…

6. Thank you for stating the following in your Competing Interests section:

"No"

Please complete your Competing Interests on the online submission form to state any Competing Interests. If you have no competing interests, please state "The authors have declared that no competing interests exist.", as detailed online in our guide for authors at <http://journals.plos.org/plosone/s/submit-now>. This information should be included in your cover letter; we will change the online submission form on your behalf.

**Response**: The following statement should be included in the online submission system:

“**The authors have declared that no competing interests exist**”.

7. In your Data Availability statement, you have not specified where the minimal data set underlying the results described in your manuscript can be found. PLOS defines a study's minimal data set as the underlying data used to reach the conclusions drawn in the manuscript and any additional data required to replicate the reported study findings in their entirety. All PLOS journals require that the minimal data set be made fully available. For more information about our data policy, please see http://journals.plos.org/plosone/s/data-availability.

Upon re-submitting your revised manuscript, please upload your study’s minimal underlying data set as either Supporting Information files or to a stable, public repository and include the relevant URLs, DOIs, or accession numbers within your revised cover letter. For a list of acceptable repositories, please see http://journals.plos.org/plosone/s/data-availability#loc-recommended-repositories. Any potentially identifying patient information must be fully anonymized.

**Response**: The following statement has been used to meet the ‘Data availability’ requirements of the journal:

“**All relevant data are within the paper and its Supporting Information files**”

**Response to Reviewer’s comments**

**Comment:** Furthermore, information should be provided about the issue of “trace positive samples” by Ultra. In addition to detection of the single-copy rpoB gene for the simultaneous diagnosis of TB and rifampicin resistance, Ultra includes two new and more sensitive PCR assays that target the multicopy IS6110 and IS1081 genes to improve M.tuberculosis detection in paucibacillary samples. Information about RIF-R is not available in IS6110 or IS1081 positive and rpoB negative samples are categorized as “trace” by Ultra. Thus MDR-TB cases can be missed among the “trace” positive samples. For these samples, information about drug resistance can only be provided by culture. Therefore, when comparing the two methods, it is important to have this information.

We have acknowledged the shortcomings of not having the data for culture and we have included it in the study limitations. However, in the literature despite the changes in the targets on IS6110 and IS1081 genes, there wasn’t a significant change in the detection of Rifampicin Resistant Cases.

Comment: Mycobacterium Tuberculosis should be written as Mycobacterium tuberculosis

We thank the reviewer for this comment, and this has been revised.

Comment:The sentence in the last paragraph of the discussion should be corrected “This study was not with limitations….”

We thank the reviewer for this comment. The authors have corrected this to “…This study was not without limitations”…
