# Supplementary material for: Comparison of tests done, and tuberculosis cases detected by Xpert® MTB/RIF and Xpert® MTB/RIF-Ultra in Uganda
Source: PLoS One. 2022 Oct 10;17(10):e0275960. doi: 10.1371/journal.pone.0275960 (PMC9550068; doi:10.1371/journal.pone.0275960)
Supplement: Attachment — Submitted filename: Response to reviewers PONE-D-20-34522R2.doc [file pone.0275960.s003.doc]

September 15^th^, 2022

The Academic Editor
PLoS ONE

Dear Academic Editor,

**RE: Response to the editor and reviewers' comments on Manuscript #: PONE-D-20-34522R2: “Comparison of tests done, and Tuberculosis cases detected by Xpert MTB/RIF and Xpert MTB/RIF Ultra in Uganda”.**

First and foremost, we would like to express our gratitude for allowing us to revise our manuscript for possible publication in your prestigious journal, *PLoS ONE.* We would also like to thank the editor and reviewers for taking the time to provide enriching scientific comments.

We have now revised the paper in line with the editor’s and reviewers’ comments and suggestions and believe that it is now a much more improved paper than the version we originally submitted.

We will be glad to provide any further explanations on the changes made in the paper, should this be necessary. Otherwise, we look forward to having our paper accepted for publication in *PLoS ONE*.

Regards

Michael Kakinda MD, MPH

Corresponding Author

**POINT-BY-POINT RESPONSE TO THE EDITOR’S AND THE REVIEWERS’ COMMENTS**

**Response to the Editor’s Comments**

The manuscript has been revised satisfactorily. However, there are two minor corrections

Line 58- 'Tuberculosis' to be replaced by 'tuberculosis'

We do thank the editor for this observation. We have made this correction.

Line 137- 'got' to be replaced by 'obtained

We have made this correction.
